# PD-L1 knockout or ZG16 overexpression inhibits PDAC progression and modulates TAM polarization

**DOI:** 10.3389/fimmu.2025.1510179

**Published:** 2025-01-31

**Authors:** Hui Meng, Manman Nan, Yizhen Li, Yi Ding, Xiaokun Fang, Weiqian Ma, Mingzhi Zhang

**Affiliations:** Department of Pathology, The First Affiliated Hospital of Zhengzhou University, Zhengzhou, China

**Keywords:** PD-L1 knockout, ZG16 intervention, CRISPR/Cas9, PDAC progression, TAM polarization

## Abstract

CRISPR/Cas9-mediated genome editing has the potential to delete PD-L1 both on the cell surface and inside the cell, thereby inhibiting tumor growth and migration and overcoming immunosuppression. ZG16, with its lectin structure, can reduce PD-L1 expression on the cell surface. However, direct comparison of two approaches on PD-L1 expression in Pancreatic ductal adenocarcinoma (PDAC) cells has not yet been investigated. In this study, we established two Panc-1 cell line: one with PD-L1 knockout and another with ZG16 overexpression. Both methods promoted the polarization of tumor-associated macrophages (TAMs) to the M1 phenotype, as indicated by increased levels of the M1 marker CD11c+ *in vitro* and *in vivo*. Meanwhile, we observed a reduction in the M2 marker CD206+, upregulation of immune activation-related cytokines/chemokines, and a decrease in immunosuppressive cytokines and tumor angiogenesis factors. In summary, both PD-L1 knockout and ZG16 overexpression represent promising approaches for PDAC treatment.

## 1 Introduction

Programmed cell death ligand 1 (PD-L1) is a member of the B7 family (also known as CD274). PD-L1 is widely expressed in malignant tumors and the amygdala, placental trophoblast cells, monocytes and lungs. PD-L1 produces an inhibitory signal for receptor-mediated T lymphocyte (T cell) activation by binding to the receptor PD-1 ex-pressed on the surface of activated T cells (1). Recent studies have shown that PD-L1 and PD-1 signaling attenuates local tumor immunity, and this immunosuppressive co-signaling (immune checkpoint) may be one of the most important components of the tumor immune tolerance mechanism (2). Clinical trials of several immune checkpoint inhibitors, such as anti-PD-1 antibodies and anti-PD-L1 antibodies, have produced significant antitumor effects in certain patients with solid tumors.

Pancreatic ductal adenocarcinoma (PDAC) is projected to surpass breast cancer and colorectal cancer, becoming the second leading cause of cancer-related mortality by 2030. One of the primary challenges associated with PDAC is its subtle clinical manifestations. Furthermore, for most patients diagnosed with advanced PDAC, the current standard treatment involves gemcitabine-based regimens or intensive chemotherapy protocols (such as FOLFIRINOX), aimed at reducing tumor volume and enhancing surgical resectability. The available clinical treatment strategies remain quite limited. Therefore, it is imperative to pursue further research to develop novel clinical approaches for treating pancreatic cancer that hold promise for long-term therapeutic responses (3).

Immunotherapy represents a relatively novel approach in the treatment of cancer, aimed at counteracting the immunosuppressive effects exerted by tumor cells (4). However, pancreatic cancer is typically regarded as a non-immunogenic tumor (5). It possesses a unique microenvironment characterized by pronounced desmoplastic re-actions, interstitial hypoxia, acidic pH levels, and other distinctive features (6). Collectively, these factors contribute to tumor development and progression while also fostering resistance to immunotherapy.

Consequently, PD-1/PD-L1 inhibitors demonstrate limited therapeutic efficacy in pancreatic cancer and may only exhibit certain benefits in select patients with microsatellite instability or deficient mismatch repair (MSI/dMMR) (7, 8).

PD-L1 is expressed on the surface of immune cells and tumor cells and binds to programmed cell death protein 1 (PD-1) to produce immune resistance. On the other hand, intracellular PD-L1 promotes cell growth, proliferation and migration. Since inhibitors and monoclonal antibodies only block cell surface PD-L1, uninhibited intra-cellular PD-L1 can still reduce the efficacy of immunotherapy. PD-L1 can be transferred from intracellular to cell surface to escape immune attack and promote the occurrence of tumor progression (9). Knockout of PD-L1 on the cell surface and inside the cell offers novel strategies with anti- PDAC therapeutic potential. Gene deletion of tumor-related genes using regularly spaced short palindromic repeats (CRISPR) and CRISPR-associated protein-9 nucleases (CRISPR/Cas9) has great potential for anticancer therapy (10).

Michael A. Curran reported that the combination of CTLA-4 and PD-1 blockers was more than twice as effective in promoting B16 melanoma as when used alone. When CTLA-4 blockers were used alone, the effect was 10%. The combination of PD-1 and CTLA-4 blocks the invasion of increased effector T cells (Teff), resulting in an increased ratio of Teff to regulatory T cells, which is highly beneficial to the tumor. Combined blocking also synergistically increases the ratio of Teff to bone marrow-derived suppressor cells in B16 melanoma. Combined blocking of PD-1/PD-L1 and CTLA-4 negative co-stimulatory pathway can make tumor-specific T cells that would otherwise be inactivated continue to expand and play an effective role, thus transforming the tumor microenvironment from an inhibitory state to an immune response state (11).

Zymogen granule protein 16 (ZG16) is a secretory glycoprotein with a Jacalin domain (a type of animal secretory lectin structure) and a Jacalin-related β- prism fold, found in the membrane of zymogen granules in the rat pancreas. It is expressed in various tissues including the pancreas, liver, small intestine, and colon. ZG16 is closely associated with the occurrence and development of various tumors. When tumor lesions develop in tissues, a notable reduction or absence of ZG16 expression can be detected (12). For instance, the expression of ZG16 is downregulated in liver cancer (13); the gene mutation of ZG16 in circulating tumor cells participates in regulating the metastasis of breast cancer (14); miR-196a can facilitate the stemness and growth of colorectal cancer by downregulating ZG16 (15). Additionally, ZG16 is significantly downregulated in colon cancer. The expression of ZG16 is conspicuously associated with molecular characteristics such as the occurrence, distant metastasis, lymphatic invasion, MLH1 gene silencing, hypermethylation status, and microsatellite instability (MSI) of colon cancer. Furthermore, the overall survival period and progression-free survival period of patients with high ZG16 expression are significantly prolonged (16) Furthermore, the injection of the ZG16 system can trigger the secretion of CD40 *in situ*, and ZG16 can collaborate with dendritic cells (DCs) to enhance their functions (17). This might offer a novel approach for the immunotherapy of pancreatic ductal adenocarcinoma (PDAC). Nevertheless, its specific mechanism of action still requires further clarification.

We also demonstrated that ZG16 promotes T-cell-mediated immunity by directly binding to glycosylated PD-L1 through its lectin domain. Overexpression of ZG16 can significantly inhibit tumor growth and enhance the efficacy of chemotherapy. Most importantly from a clinical point of view, ZG16 can be activated as a small molecule protein delivery drug to block the expression of PD1 gene and CTLA4 expression in T cells *in vivo*. It is a novel multi-target immune checkpoint inhibitor (12–14).

Meanwhile, overexpression of ZG16 in colon cancer cells significantly influences the expression of both stimulatory and inhibitory checkpoint molecules, including CD40, PD1, and CTLA4 (18), promotes the secretion of pro-inflammatory mediators, and enhances T-cell-dependent antitumor effects (19).

It is evident that ZG16, to a certain extent, exerts the immune modulatory function of PD-1/PD-L1 inhibitory antibodies. The mechanisms of its action on CD8+T cells, dendritic cells, etc., need to be further clarified through experiments. We also anticipate more extensive clinical data to confirm its role in tumor immune modulation and further explore new immunotherapeutic approaches based on this. We attempted to compare the effect of PD-L1 knockout (KO) with the overexpression of ZG16 to reveal whether there is a deeper genetic connection between these two pathways or whether there are overlaps or differences in signaling pathways between them.

To compare its targeting of immune checkpoints, we used Crispr to knock out PD-L1and overexpressed ZG16 in human PDAC cell line Panc-1, and compared the cell functions of the two different treatments. Bioinformatics methods were employed to analyze the alterations in the transcriptome gene profile of human PDAC cells following the two biological interventions. Thus demonstrating the biological role of the two interventions in PDAC. Further, animal-derived PDAC cells were used to knock out PD-L1 and overexpress ZG16 respectively. By comparing the *in vivo* inhibition of PDAC and the effects of the two biological interventions on the immune microenvironment of PDAC, the therapeutic effects of PD-L1 alone targeted therapy and ZG16 multi-targeted therapy on PDAC were clarified. In order to seek a new strategy for clinical treatment of PDAC.

## 2 Materials and methods

### 2.1 Preparation of cas9-gRNA lentiviral vector (Panc-1 PDL1)

The human pancreatic cancer cell line Panc -1 was purchased from the American Type Culture Collection (ATCC,CRL-1469)

#### 2.1.1 Construct information: pHBLV-U6-gRNA-EF1-CAS9-PURO

#### 2.1.2 h-CD274-gRNA sequence: 5’ -tctttatattcatgacctac -3’

#### 2.1.3 Synthesis of primers

Primers were synthesized PAGE-purified oligo sequences from Bio and diluted to 100uM, respectively. The system is as follows (20ul):10 *oligo Buffer: 2uL; sgRNA-F: 1uL; sgRNA-R: 1uL; H_2_O: 16uL. Annealing procedure: 95°C, 10min; 75°C, 10min; 55°C, 10min; 35°C, 10min; 15°C, 10min.

#### 2.1.4 Vector digestion

Each reagent was added in turn, gently sucked and mixed, and the reaction was placed in a 37°C water bath for 1-2h. After digestion, agarose gel electrophoresis was carried out to recover the target fragment. The vector digestion system is as follows: vector DNA (1ng/ul) 1ul; 10 × buffer 4ul; ddH_2_O 32ul; restriction enzyme 1; 1.5ul; restriction enzyme 2; 1.5ul; total 40ul.

#### 2.1.5 Connection between interference fragment and carrier.

The connected reaction system (20uL) is as follows: annealing product 1ul; enzyme cut carrier X (≥50ng); T4 ligase buffer 2ul; T4 ligase 1ul; H_2_O 16-X; total 20ul. Connections were made overnight at 16°C.

#### 2.1.6 Transformation

DH5α competent cells were taken out of the -80°C refrigerator and immediately put on ice to melt. After being competent melt, with 50 uL volume per tube packing, 5μL of ligation product was added. Leave on ice for 20-30min; 42°C heat shock strict control in 90 seconds. After heat shock, it was immediately inserted into the ice for 2-3 minutes. In the ultra-clean table, 500μL LB medium without antibody was added and gently reversed up and down for 3-5 times. Shock culture at 230rpm at 37°C for 45-60 min. The bacteria solution was coated on the corresponding resistant solid plate, evenly coated, and then the plate was put upside down in a 37°C incubator for 12-16h.

#### 2.1.7 Bacterial liquid PCR identification

PCR identification system of bacterial solution: 2xHieff PCR master MIX (Dye) 5μL; primer 1, 0.5μL; primer 2, 0.5μL; bacterial solution 2μL; ddH_2_O 2μL; Total volume 10μL. Microbial PCR identification procedures: Pre-denaturation 94°C5 min 1cycle; Denatured 94°C 30 sec, Anneal 56°C 30 sec, Extend 72°C 30~60 sec/kb, 25cycles, thoroughly extend 72°C 10 min 1cycle, save for 12°C.

#### 2.1.8 Plasmid transfection

Sequencing, h-CD274 sgRNA1 sequencing results are as follows: 5’-TCTTTATATTCATGACCTAC -3’. Plasmid extraction: after successful sequencing, the bacterial solution was amplified according to the project requirements, and the plasmid extraction and purification were carried out. The plasmid extraction scheme was subject to the instructions of the extraction kit. The extracted plasmids were verified by QC and then transfected into cells.

### 2.2 Transcriptome sequencing-RNA extraction and library construction

Total RNA was extracted by TRIzol reagent according to the instructions. RNA purity and quantification were determined using a NanoDrop 2000 spectrophotometer (Thermo Scientific, USA). RNA integrity was assessed using the Agilent 2100 Bioanalyzer (Agilent Technologies, Santa Clara, CA, USA). The transcriptome Library was constructed using the VAHTS Universal V5 RNA-seq Library Prep kit as instructed.

### 2.3 Transcriptome sequencing analysis methods: RNA sequencing and differential expression gene analysis

The constructed transcriptome sequencing library qualified for quality control was sequenced by llumina Novaseq 6000 sequencing platform, and 150 bp double-ended reads were obtained. Approximately 20 million raw readings were obtained for each sample. The original reads were processed in fastq format using Fastp software, and clean reads were obtained after removing low-quality reads for subsequent data analysis. HISAT2 software was used for reference genome comparison and gene expression count (FPKM) was calculated. Reads of each gene were obtained by HTSeq-count (counts). PCA analysis and mapping of genes (counts) using R (v 3.2.0) to assess sample biological duplication. DESeq2 software was used to analyze differentially expressed genes, in which genes meeting the threshold of q < 0.05 and foldchange > 2 or foldchange < 0.5 were defined as differentially expressed genes (DEGs). R (v 3.2.0) was used for hierarchical cluster analysis of DEGs to show the expression patterns of genes in different groups and samples. R-package ggradar was used to make radar map of the top 30 genes to show the up-regulated or down-regulated gene expression changes. Subsequently, GO, KEGG Pathway, Reactome and WikiPathways enrichment analysis of differentially expressed genes were conducted based on hypergeometric distribution algorithm to screen the items of significant enrichment function. R (v 3.2.0) was used to draw column diagram, chord diagram or enrichment analysis circle diagram for the items of significant enrichment function.

### 2.4 To investigate the impact of PD-L1 silencing and ZG16 overexpression on immune cells in a murine model of PDAC mice

A silent lentivirus based on mouse PD-L1 (LV-shPD-L1) and negative control (LV-shNC) was constructed. Mouse ZG16 (NM_026918.3, CDS 504 bp) was used to construct overexpressed lentivirus and negative control.

Model building: All animal surgeries were approved by the Animal Care and Use Ethics Committee of the First Affiliated Hospital of Zhengzhou University. Eight-week-old female C57BL/6J mice were intraperitoneally injected with 1×10^5^ cells (total volume of 1ml) to establish an intraperitoneal metastasis model. After a period of 14 days, the mice were euthanized, and the number of tumors in the abdominal cavity was observed and documented. Tumor tissues were collected and weighed. Ascites was collected for measurement of its volume. The expression levels of CD4 (CD4+T cells), CD8 (CD8+T cells), CD49 (NK cells), xzPD-1, PD-L1, and CTLA4 in tumor tissues were assessed using immunohistochemistry, followed by analysis of corresponding immune cell populations. Immunofluorescence double staining was employed to examine co-localization patterns between F480 and CD11c, F480 and CD206, F480 and CXCL9, as well as F480 and CXCL10 in tumor tissues. Real-time PCR was conducted to determine mRNA expression levels of IFN-γ (IFNG), TNF-α, IL-2, IL-12a, IL-10, VEGFa, CXCL9, CXCL10, CXCL1, and CXCL2 in tumor tissues. Furthermore, the expression of IFN-γ, TNF-α, IL-10, and VEGF in ascites was measured using ELISA.

### 2.5 Flow cytometry

Flow cytometry was employed to determine the proportion of tumor-infiltrating lymphocytes in each experimental group. Tissues were incubated at 37°C for 45 minutes in RMI-1640 medium supplemented with 1 mg/ml IV collagenase type and 0.2 mg/ml dnase I type. Subsequently, the resulting solution was filtered through a 70 mm filter to obtain a single-cell suspension. Fixed survival stain 510 (BD Bioscience) was utilized for survival staining and FcgR blockage. CD11c antibody (flow, IF, anti-human/mouse; different species origin from F480 antibody; BD Bioscience) and CD206 antibody (IF, anti-CD206 antibody; anti-mouse; different from F480 antibody; BD Bioscience) were used for immunostaining purposes. The cell suspension was stained accordingly. For apoptosis analysis, cells were washed and resuspended in buffer (2*10^6^), followed by double-staining with AnnexinV-Light650 (5 μl) and Propidium Iodide (10 μl) to detect apoptotic events. Two tubes of single-stained positive control samples were kept in darkness for 15 minutes prior to analysis using a BD LSR Fortessa flow cytometer (BD Biosciences). Data analysis was performed using FlowJo software (Tree Star).

### 2.6 Double-color immunofluorescence analyses

The infiltration of macrophage subtypes in tumor tissues was assessed using anti-CD11c, anti-CD206, and rat anti-F480 antibodies. FITC-conjugated secondary antibodies (Jackson Immuno Research) were employed to visualize CD11c-positive and CD206-positive cells, while F480-positive cells were visualized using Cy3-conjugated secondary antibodies (Jackson Immuno Research, West Grove, PA, USA). Nuclei were counterstained with DAPI. Similar immunofluorescence assays were conducted utilizing anti-CXCL9 and anti-CXCL10 antibodies. Fluorescent signals were observed under a fluorescence microscope (BZ-X700; Keyence, Osaka, Japan).

Cell culture and transfection. Panc-1 cells (ATCC, Manassas, VA) and Panc02 cells were cultured in growth medium containing 1640 (Sigma-Aldrich, St Louis, MO) supplemented with 10% fetal bovine serum (FBS; Sigma-Aldrich, St. Louis, Missouri) and 1% penicillin-streptomycin (Thermo Fisher Scientific, Waltham, MA) were cultured at 37°C and 5% CO2.

### 2.7 Western blot

Cells were washed twice in PBS and lysed with RIPA buffer containing 1X protease inhibitor mixture (Thermo Fisher Scientific, Waltham,MA), or the cells were lysed with Abcam nuclear extraction kit (Cambridge, United Kingdom). Protein extracts were collected and quantified using Pierce BCA protein detection kit (Thermo Fisher Scientific, Waltham, MA). Twenty µg of protein was loaded onto 12% SDS-PAGE gel (Bio-Rad Laboratories, Hercules, CA) and run at 100 V for 1.5 hours. Samples were transferred to nitrocellulocellulosic membranes (Bio-Rad Laboratories, Hercules,CA) and checked by Ponceau staining (Sigma-Aldrich, St Louis, MO). Blots were washed in PBS to remove the staining, then blots were blocked in 3% milk (Bio-Rad Laboratories, Hercules, CA). The imprint was stained with rabbit PD-L1 (1:500; Thermo Fisher Scientific Inc., Waltham, MA), rabbit beta-actin (1:500; Thermo Fisher Scientific, Waltham, Mass.), rabbit Flag (1:500; Cell Signaling Technology, Danforth, Mass.) or rabbit histone H3 (1:500; Novus Biologicals, Littleton, CO) in 3% milk overnight at 4°C. The blots were cleaned in PBST (0.25% Tween-20 in PBS) and coated in secondary antibodies (rabbit IgG, enzyme-conjugate antibody; 1:2000. Cell Signaling Technology, Danvers, MA)1 hour. The samples were washed in PBST and imaged on the Amersham Imager 680 Imprinting and gel Imager (GE Healthcare, Chicago, IL). The blotches were quantified using Image Pro Plus software (Media Cybernetics, Bethesda, MD) and the data were normalized for direct comparison using the formula x ‘= x−µσ, where x = data value, µ= mean value of the data set, and σ = standard deviation.

### 2.8 Co-culture with human macrophages

THP-1 was cultured normally in RPMI 1640 medium supplemented with 10% fetal bovine serum. After treated with 100 ng/mL PMA for 24 h, THP-1 cells were induced to differentiate into M0 macrophages. Panc-1 cells and M0 macrophages were co-cultured by Transwell non-contact co-culture system. 5×10^5^ Panc-1 cells transfected with ZG16 overexpression plasmid, PD-L1 knockout Panc-1 cells and blank control cells were pre-seeded in the upper chamber of Transwell one day in advance. 5×10^5^ M0 macrophages were cultured in the inferior cavity. On the second day, the upper cavity was moved to the lower cavity with 6 well plates for co-culture. After 48 h of co-culture, macrophages from the inferior lumen were collected as tumor-related macrophages (TAMs). The expression of CD11c and CD206 in TAM cells was detected by flow cytometry. The levels of IL-4 and TNF-α in the culture medium were detected by ELISA.

### 2.9 Enzyme−linked immunosorbent assay

The levels of IL-4 and TNF-α in the supernatant of culture medium and the expressions of IFN-γ, TNF-α, IL-10 and VEGF in ascites were detected by ELISA. Co-culture cell supernatants were detected using an enzyme-linked immunosorbent assay (ELISA) and cytokine secretion was detected using an enzyme-linked immunosorbent assay kit (Thermo Fisher Scientific, Waltham, MA). ELISA follows the manufacturer’s protocol. Data were collected at 450 nm and 570 nm on the FlexStation 3 multimode Microplate Reader (Molecular Devices, San Jose, CA).

### 2.10 Immunohistochemical methods

Thin sections of paraffin-embedded tissue, approximately 3μm thick, were prepared. Following baking, wax removal, and dehydration steps, the tissues underwent microwave treatment at 100°C for 10 minutes using a 0.01M citrate buffer (pH 6.0). To block endogenous peroxidase activity, a solution containing 3% H_2_O_2_ was applied to the tissues followed by blocking with normal sheep serum. Primary antibodies were then added and incubated overnight at 4°C in a refrigerator. After cooling to room temperature, cells were washed three times for five minutes each with PBS buffer. Subsequently, a biotin-labeled secondary antibody derived from sheep rabbit was introduced and allowed to incubate at room temperature for ten minutes before being washed again with PBS buffer three times for five minutes each time. The addition of an hrp-labeled working solution ensued and incubation took place at 37°C for ten minutes prior to another round of washing with PBS buffer three times for five minutes each time. This was followed by rinsing with PBS buffer three more times for five minutes each time.

To visualize the stained tissues under a microscope and record the staining results, DAB/H2O2 staining was performed on the specimens’ sections after which they were treated briefly with reverse hematoxylin (two-minute exposure), differentiated (one-second duration), washed thoroughly with water (two-minute rinse), subjected to blue restoration using PBS solution, dehydrated through gradient alcohol treatment, dealcoholized via xylene immersion, and finally sealed using neutral glue.

### 2.11 CCK-8 test

In the 96-well plate, 100μl of 5000 cell/ml cell suspension solution culture plate was prepared in the incubator for 24 hours, 37°C, 5% CO2, 10μl CCK8 solution per well, and continued to incubate for 1-4 hours. The absorbance at 450nm was measured using a microplate reader MTT assay.

### 2.12 Invasion and migration studies

Each 24-well plate was inoculated with 100,000 cells and transfected 24 hours later. After 48 h, the cells were scratched, and at 0, 8, and 24 h after scratching, the invasion of cells to the scratch surface was observed using an EVOS digital inverted fluorescence microscope (Thermo Fisher Scientific, Waltham, MA). The cells were counted and the data normalized for direct comparison throughout the experiment. The inhibitory effects of PD-L1 knockout and ZG16 overexpression on Panc-1 cell migration were detected by a double-layer insertion culture system. The cells were spread on an insert sheet in a 3µm well, and the cells in each group were treated for transfection. Cells were observed to migrate from the top layer of the insertion plate to the bottom layer of the plate. The number of Panc-1 cells migrated to the bottom layer was counted under the microscope at day 1, 2 and 3 respectively. The cell proliferation level of each group was detected by CCK-8 kit at 0, 24, 48 and 72 h, respectively. The apoptosis level of each group was detected by flow cytometry.

### 2.13 Statistical analysis

SPSS (IBM, Armonk, NY) software was used for statistical analysis. The t test was used for comparison between the two groups, and the measurement data were expressed as mean ± standard deviation (SD). One-way analysis of variance and Hoc analysis for multiple comparisons were performed using Tukey’s post. A P value of < 0.05 was considered statistically significant. The variance was similar between groups, and the data conformed to a normal distribution. All experiments were performed in triplicate and data were normalized for direct comparison.

## 3 Results

### 3.1 Establishment of a system for identifying CRISPR/Cas9 plasmids containing dual sgRNAs and template synthesis for HDR

**Figure 1:**
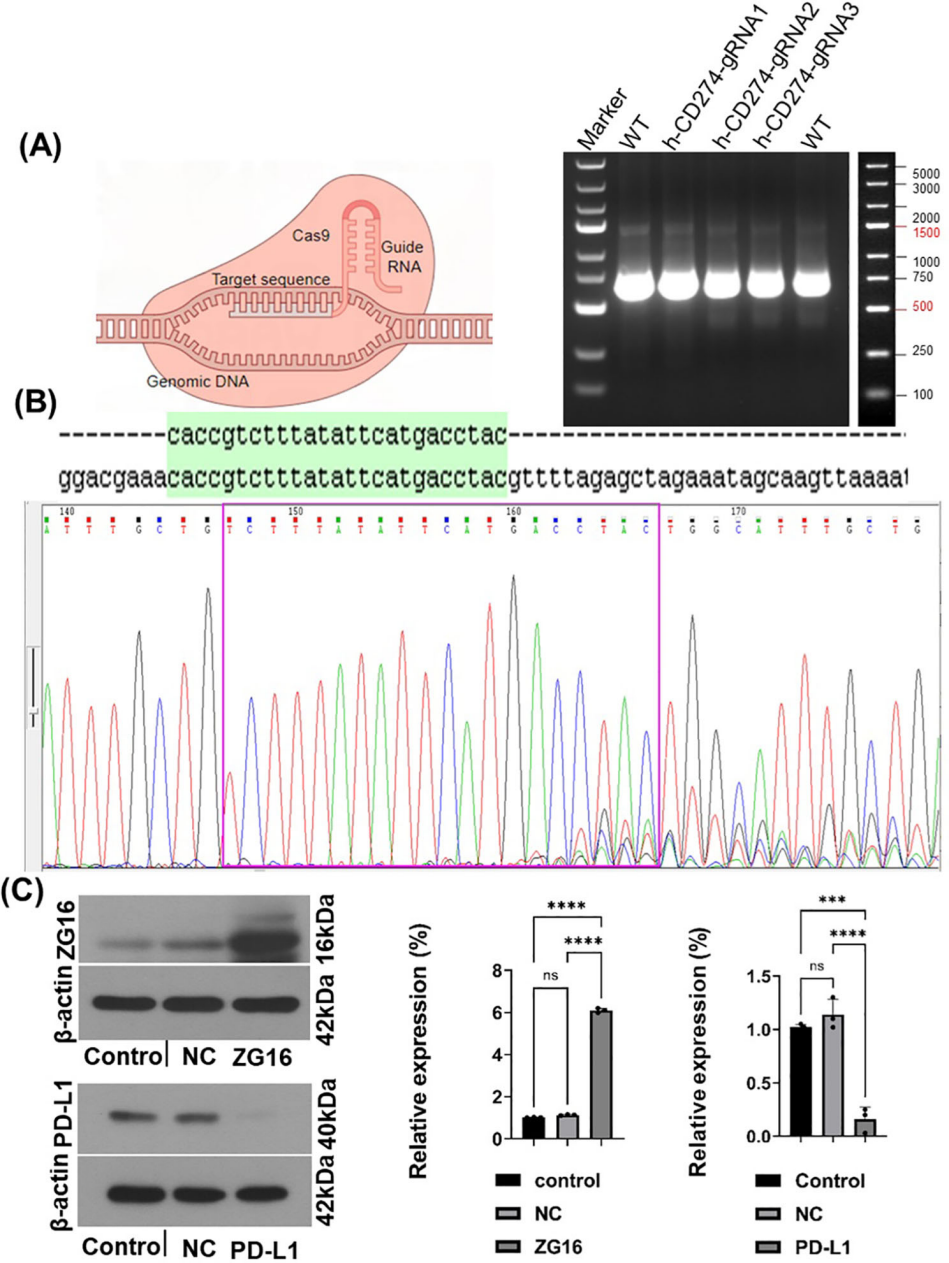
The establishment of human PDAC cells with PD-L1 knockout and ZG16 overexpression was achieved. **(A)** The PD-L1 gene was completely deleted in Panc-1 PDAC cells using the Cas9 gene editing technique. Genomic PCR results, and the genome size between PCR primers was 619bp. **(B)**. h-CD274 gRNA sequencing, (green region is the target sequence). **(C)** Efficacy verification of PD-L1 knockout and ZG16 overexpression (western blot), Statistical analysis was performed using one –way ANOVA, (***p < 0.001, ****p < 0.0001). ns, not statistically significant.

We used the CRISPR/Cas9 system to generate a stable PDAC knockout cell line (**Supplementary Figure 1**). To evaluate the efficacy of PD-L1 knockout, we employed the T7E1 assay. This assay involves primer amplification of a specific genomic DNA region encompassing the target site, followed by denaturation, reannealing, and cleavage using T7 endonuclease. Panc-1 cells were transfected with h-CD274-sgRNA1, h-CD274-sgRNA2, and h-CD274-sgRNA3. Successful gene editing in Cas9-h-CD274-sgRNA3 -treated cells was confirmed through PCR (**Figure 1A**). The transfection efficiency was confirmed by sanger sequencing (**Figure 1B**). Furthermore, we overexpressed ZG16 by lentivirus in Panc-1 cells (**Supplementary Figure 1**). The effect of PD-L1 knockout and ZG16 overexpression was assessed using western blot analysis (**Figure 1C**).

### 3.2 Results of RNA sequencing

**Figure 2:**
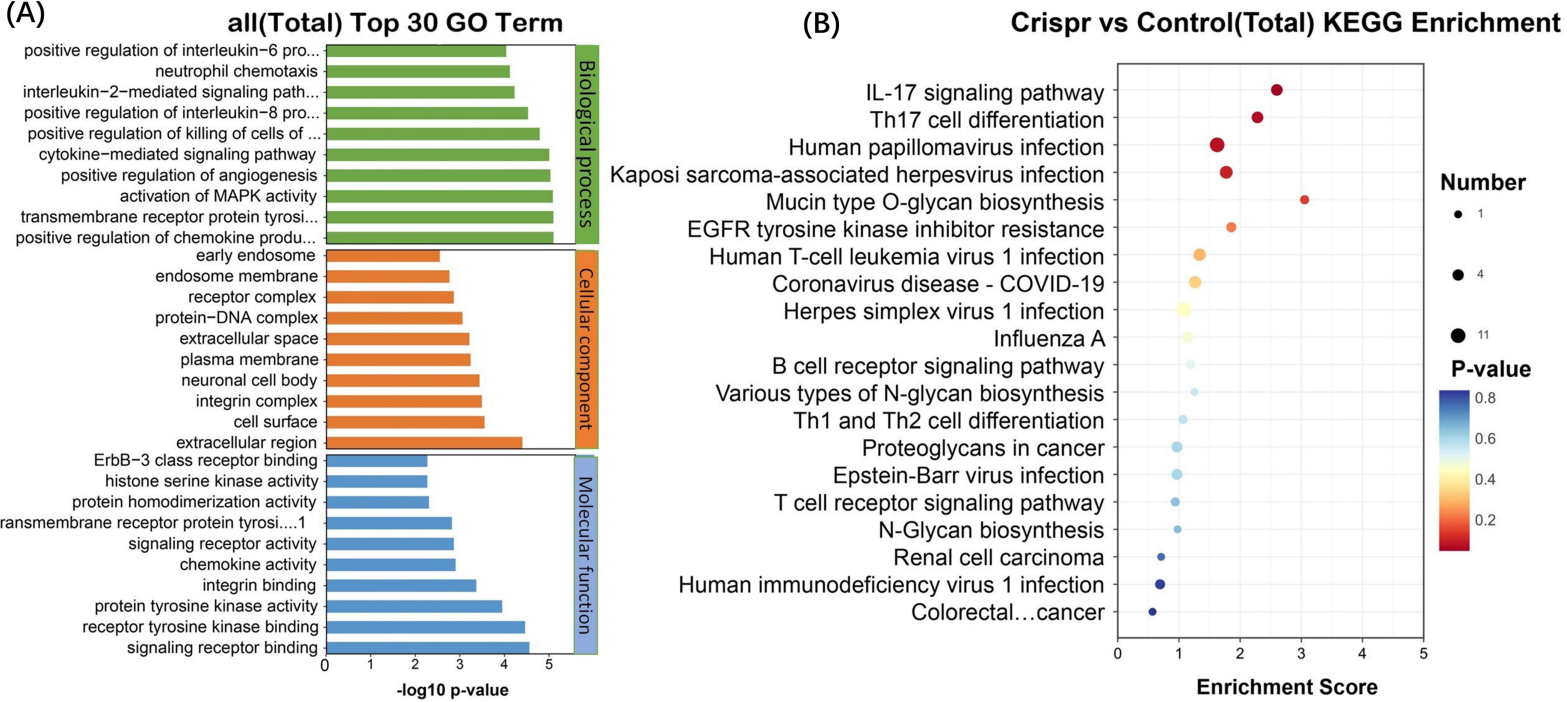
The significantly different genes after PD-L1 gene was knocked out by CRISPR technology were obtained by RNA sequencing. **(A)** Functional pathways involved: allograft rejection, Alzheimer disease, AMPK signaling pathway, Amyotrophic lateral sclerosis, Autoimmune thyroid disease, B cell receptor signaling pathway, Central carbon metabolism in cancer, Chemokine signaling pathway, Coronavirus disease - COVID-19, ECM-receptor interaction, EGFR tyrosine kinase inhibitor resistance, ErbB signaling pathway, Glycerolipid metabolism, Glycerophospholipid metabolism, Glycosaminoglycan biosynthesis - chondroitin sulfate**/**dermatan sulphate, Glycosphingolipid biosynthesis - globo and isoglobo series, Glycosylphosphatidylinositol (GPI)-anchor biosynthesis, Graft-versus-host disease, Herpes simplex virus 1 infection, HIF-1 signaling pathway, Human immunodeficiency virus 1 infection, Human papillomavirus infection, Human T-cell leukemia virus 1 infection, IL-17 signaling pathway, Influenza A, Mannose type O-glycan biosynthesis, MAPK signaling pathway, Mucin type O-glycan biosynthesis, Natural killer cell mediated cytotoxicity, N-Glycan biosynthesis, Proteoglycans in cancer, Renal cell carcinoma, Th17 cell differentiation. **(B)** KEGG enrichment and functional top20 bubble map, the horizontal axis of the figure is the enrichment score. The larger the bubble, the more the number of differentially expressed protein-coding genes. The color of the bubble changed from blue-white-yellow-red, and the smaller the enrichment p-value value, the greater the significance. The ordinate is the pathway and the abscissa is the enrichment score.

RNA sequencing was used to obtain significant differential genes after knockout of PD-L1 gene by CRISPR technology, and the significant functional pathways obtained included immunogenic diseases and immunomodulation-related pathways. These include graft-versus-host immunity, autoimmune diseases, viral immunity, cancer metabolism, chemokine pathways, extracellular receptors, tyrosine kinase pathways, oncogenes, glycoproteins, aging, stem cells and other pathways (**Figure 2A**). Twenty significant differentially related genes were identified by KEGG (**Figure 2B**) enrichment.

**Figure 3:**
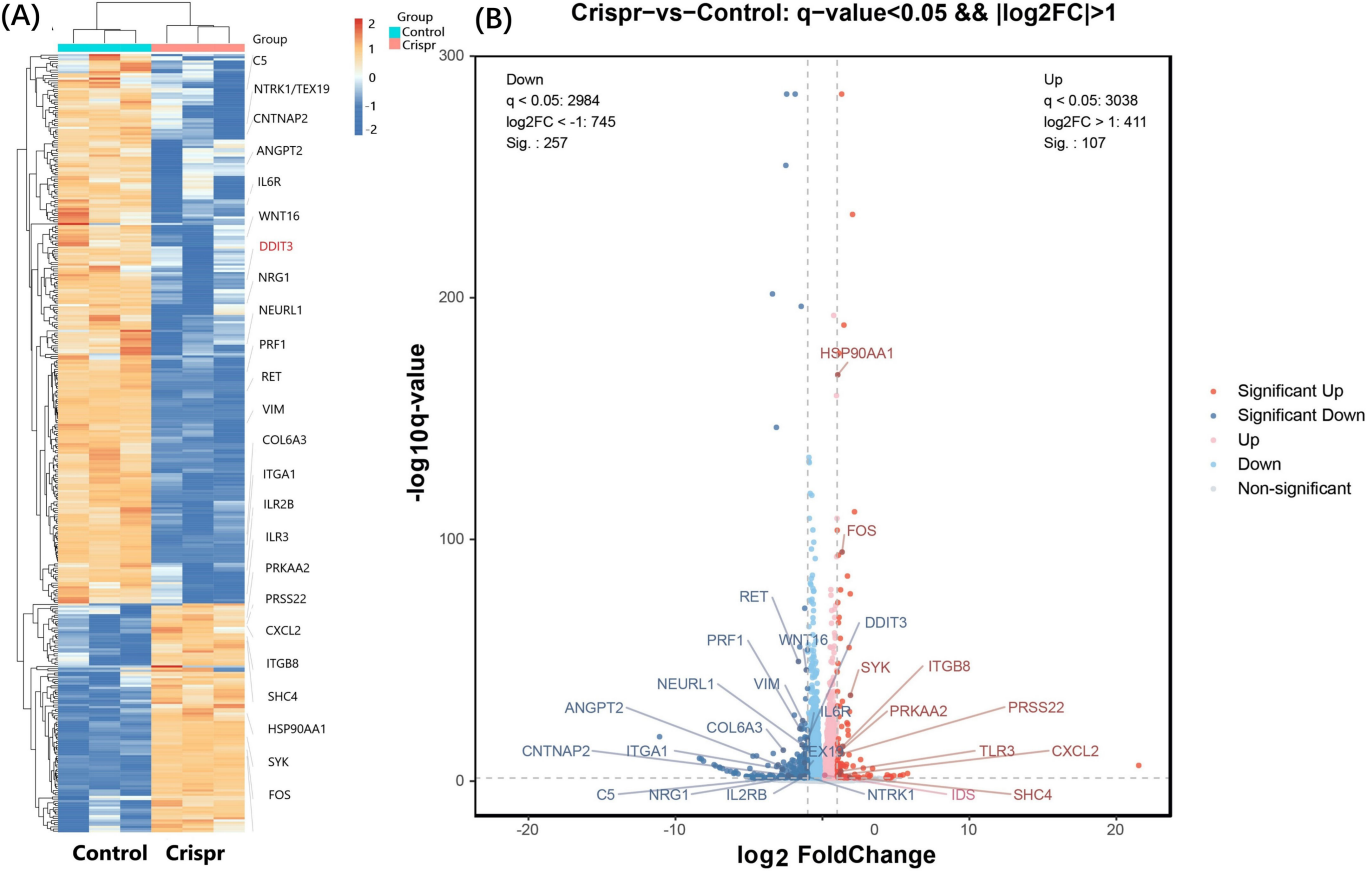
Significant differentially expressed genes were identified through RNA sequencing. **(A)** A total of 20 differentially expressed genes (focusing on tumor immunity, glycosylation and viral infection) were obtained after PDL1 knockout by CRISPR. The differentially expressed genes after PDL1 knockdown were verified in ZG16-overexpressing cells. Six genes (NEURL1, DDIT3, VIM, TEX19, PRSS22, CNTNAP2) were found to be differentially expressed in the two cell lines. Heat map annotation of 20 PDL1 knockout differential genes plus 6 PDL1 knockout/ZG16 overexpressed cross genes, a total of 25 genes were annotated after removing duplication, among which DDIT3 gene was specially annotated. DDIT3 gene expression was increased in overexpressed ZG16 cells, while its expression was decreased in PDL1 knockout cells. **(B)** After removing the duplication, the difference of a total of 25 genes was marked by volcano plot: the differences generated by comparison were reflected in the volcano plot to show the overall distribution of differentially expressed genes. The gray was the genes with non-significant differences, and the red and blue were the genes with significant differences. The horizontal axis is log2FoldChange, and the vertical axis is -log10q-value. The higher the horizontal axis is, the greater the difference is, and the higher the vertical axis is, the stronger the significance is. Blue: significantly down-regulated differentially expressed genes, red: significantly up-regulated differentially expressed genes, light blue and light red are genes filtered out by Fc threshold, grey: non-significantly differentially expressed genes filtered out by q value.

After Crispr knockout of PDL1 gene, 20 differentially expressed genes (related to tumor immunity, glycosylation and viral infection, etc.) were obtained. The differentially expressed gene after PDL1 knockout was verified in ZG16- overexpressed cells. Six differential genes were found to occur simultaneously in two cell lines (**Figure 3A**). Heat map annotation 20 differential genes +6 cross genes knocked out in PDL1 (**Figure 3B**), a total of 25 genes were annotated after removing duplicates, among which DDIT3 gene was specially annotated. DDIT3 gene expression was increased in overexpressed ZG16 cells and decreased in PDL1 knockout cells.

### 3.3 The absence of PD-L1 and overexpression of ZG16 can prevent the invasion and migration of Panc-1 cells

**Figure 4:**
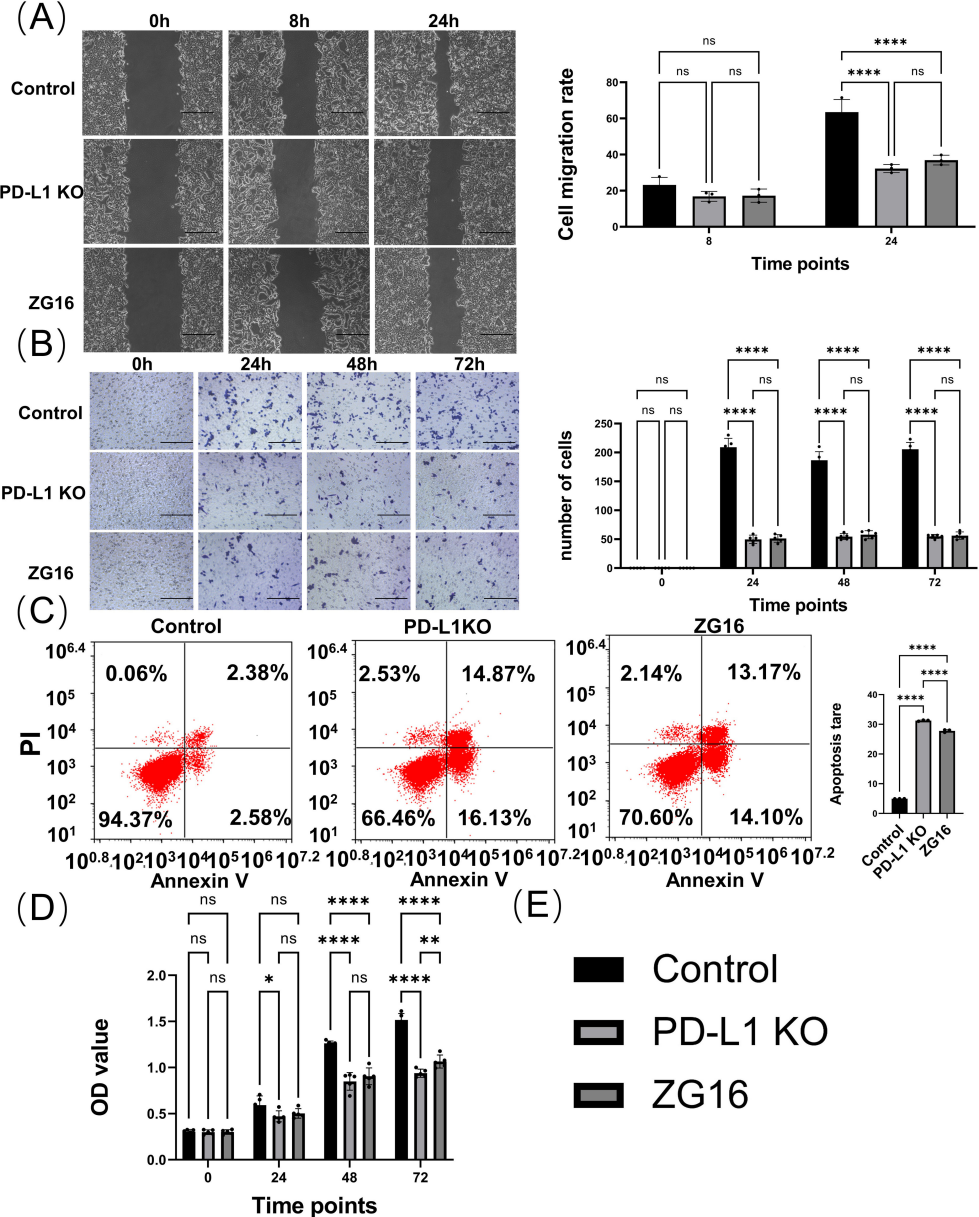
*In vitro* evaluation of the effects of PD-L1 knockout and ZG16 overexpression on Panc-1 cell functionality. **(A)** Cell scratch assay evaluated the invasion capacity of Panc-1cells. Scale bar=100 µm. The experimental results were replicated at least three times, followed by the application of standardized quantitative statistics. Cell scratch, was analyzed using two-way ANOVA. **(B)** Transwell assay assessed cell migration ability with a 3-day observation period. Scale bar=100 µm. Migration was analyzed using two-way ANOVA, while **(C)** PD-L1 knockout and ZG16 expression induced apoptosis in the cells. Cell apoptosis was assessed using one-way ANOVA. **(D)** Knockdown of PD-L1 and overexpression of ZG16 suppressed Panc-1 cell proliferation. Proliferation was analyzed using two-way ANOVA. **(E)** Statistical clustering. *p < 0.05, **p < 0.01, ****p < 0.0001, ns, not statistically significant.

Panc-1 cells were seeded in 12-well plates, treated with Cas9 + HDR and ZG16 overexpression, and incubated for 24 h prior to scratch test. Invasion of Panc-1 cells in the scratch area for 8h, 24h (**Figure 4A**, **Supplementary Figure 1**). Compared with the control group, Cas9 + HDR treatment and overexpression of ZG16 deletion prevented Panc-1 cell invasion (**Figures 4A, E**). The inhibitory effects of PD-L1 knockout and ZG16 overexpression on Panc-1 cell migration were detected by a double-layer insertion culture system (**Supplementary Figure 1**). The cells were spread on an insert sheet in a 3µm well, and the cells in each group were treated for transfection. Cells were observed to migrate from the top layer of the insertion plate to the bottom layer of the plate. The number of Panc-1 cells migrated to the bottom layer was counted under the microscope at day 0, 1, 2 and 3 respectively. In Panc-1 cells, PD-L1 knockout and ZG16 overexpression resulted in significantly reduced cell migration (**Figures 4B, E**). Depletion of intracellular and surface PD-L1 and overexpression of ZG16 induced apoptosis in the cells of PDAC Panc-1 (**Figures 4C, E**). The CCK-8 kit was used to detect the proliferation levels of cells in the blank control, PD-L1 knockout, and ZG16 expression groups at 0, 24, 48, and 72 h of culture (**Figures 4D, E**, **Supplementary Figure 1**). **Figure 4E**: statistical clustering.

### 3.4 PD-L1 knockout and ZG16 overexpression regulates TAM polarization

**Figure 5:**
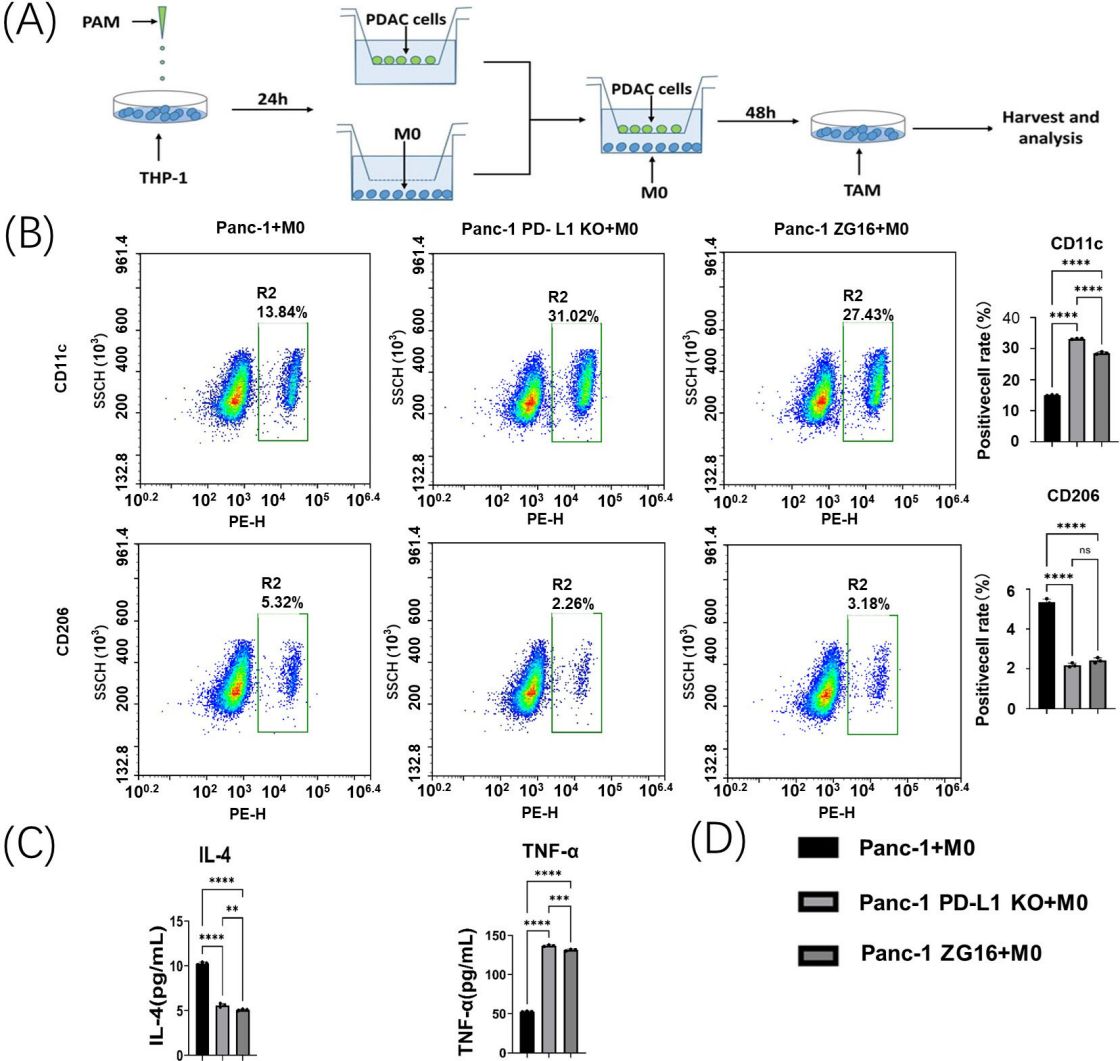
Panc-1 macrophage polarization assay *in vitro.* **(A)** Co-culture system of Panc-1 cells and M0 macrophages. **(B)** Flow cytometry was used to detect changes in the polarization of co-incubated tumor-associated macrophages (TAMs) by Panc-1 cells after PD-L1 knockdown and ZG16 overexpression. Statistical analysis was performed using one-way ANOVA, ****p < 0.0001. **(C)** The levels of IL-4 and TNF-α in the supernatant of the co-incubation medium were measured by ELISA. Statistical analysis was performed by one-way ANOVA, **p < 0.01, ***p < 0.001, ****p < 0.0001. **(D)** Statistical clustering. ns, not statistically significant.

In order to investigate the effect of Cas9-h-CD274-KO gRNA knockout of PD-L1 and overexpression of ZG16 on PDAC immune microenvironment, Panc-1 cells were co-cultured with human macrophage cell lines to establish an *in vitro* PDAC model (**Figure 5A**). co-culture system was divided into three groups. The first group consisted of Cas9-h-CD274-KO gRNA pre-treated Panc-1 cells with PD-L1 knocked out for 24 h; the second group consisted of Panc-1 cells pretreated with ZG16 overexpression for 24 h; the third group consisted of untreated Panc-1 cells co-cultured with macrophages (NC). 24 h later, macrophages were added and co-cultured to observe TAM differentiation.

The TAM polarization of Panc-1 cells in each group was detected by flow cytometry. CD11c+ M1 labeling was upregulated in co-culture with PD-L1-null and ZG16-overexpressing Panc-1 cells (**Figures 5B, D**). In contrast, their CD206 + M2 TAMs were significantly reduced (**Figures 5B, D**).

This change in M2/M1 suggests that PD-L1 loss and ZG16 overexpression in Panc-1 cells can up-regulate M1 TAMs and down-regulate M2 populations in an *in vitro* PDAC environment.

### 3.5 The deletion of PD-L1 and overexpression of ZG16 changed the secretion of inflammatory factors by TAMs co-cultured with Panc-1 cells

The effects of PD-L1 knockout and ZG16 overexpression on functional polarization of TAMs in Panc-1/macrophage co-culture system were examined. Co-culturing human macrophages with PD-L1 knocked out- or ZG16 overexpressed- Panc-1 cells resulted in a significant reduction of soluble IL-4 (M2 marker) levels in the cell culture medium, as measured by ELISA (**Figures 5C, D**). Conversely, the expression of TNF-α (M1 marker) was significantly increased in the PD-L1 knockout and ZG16 overexpression groups (**Figure 5C**). These findings suggest that the PD-L1 knockout and ZG16 overexpression in PDAC cells directly modulate the polarization of TAMs. The ablation of PD-L1 gene and up-regulation of ZG16 gene in Panc-1 cells can enhance the tumor microenvironment, offering a potential therapeutic strategy for PDAC. **Figure 5D**: statistical clustering.

### 3.6 Knockout of PD-L1 and overexpression of ZG16 impede the progression of intra-peritoneal disseminated PDAC in mice

**Figure 6:**
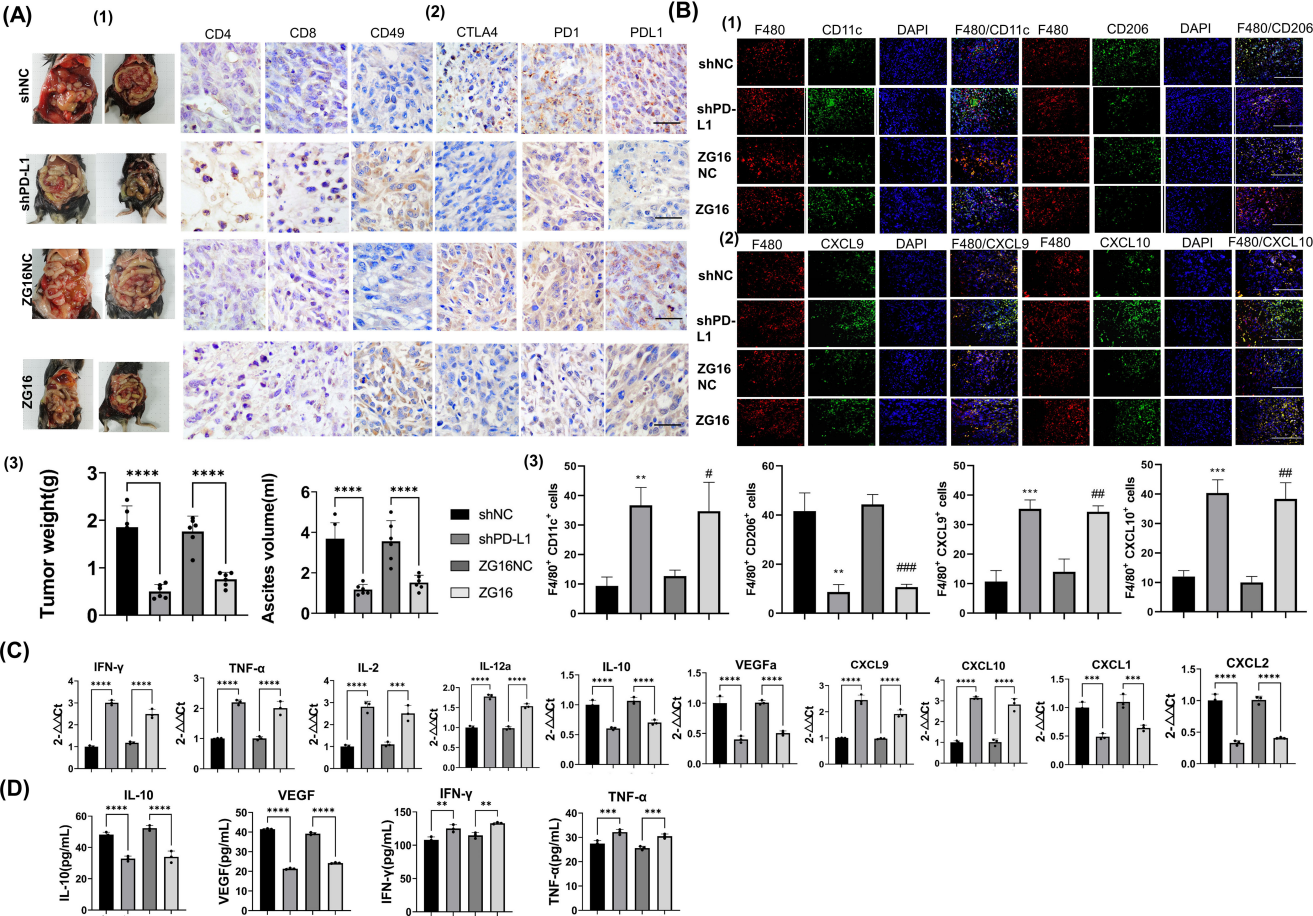
To investigate the impact of PD-L1 silencing and ZG16 overexpression on immune cells in tumor tissues and ascites within a murine model of PDAC. **(A)**. To investigate the impact of programmed cell death ligand 1 (PD-L1) knockdown and ZG16 overexpression on tumor dissemination in mice, we inoculated shPD-L1 (PD-L1-KO), ZG16 overexpression (ZG16), and control (shNC, ZG16NC) cells into mice. After a 14-day period, the mice were sacrificed to evaluate the number of tumors and ascites volume in the abdominal cavity **(A**-(1/3)). On day 14, we assessed the cellular abundance within the tumor microenvironment of PD-L1 knockout group, ZG16 overexpression group, and control group. Compared to the control group, up-regulation was observed in CD4+ T cells, CD8+ T cells, and natural killer (NK) cells’ expression levels (CD49) in both PD-L1 knockout group and ZG16 expression group; meanwhile down-regulation was observed in PD-1 + immune cells’, CTLA4+ cells’, and PD-L1 + cells’ expression levels **(A**-(2)). Scale bar=20 µm. Disruption of programmed cell death ligand 1(PD-L1) or overexpression of ZG16 within tumors enhances antitumor immunity by augmenting tumor-infiltrating lymphocytes. All values are presented as mean ± standard error (n = 6). One-way analysis of variance was used for statistical analysis **(A**-(3)). ****p <0.00001 **(A**-(3)). **(B)** Additionally, immunofluorescence double staining was employed to examine the co-localization of F480 with CD11c (F480/CD11c) 、CD206 (F480/CD206) 、CXCL9 (F480/CD206) 、CXCL10 (F480/CXCL10) in macrophages respectively **(B**-(1-2)). M1 was characterized by CD11c positivity while M2 was indicated by CD206 expression. Macrophages were stained red using an F480 antibody, while nuclei were stained blue. The markers CD11c, CD206, CXCL9, and CXCL10 were stained green. Images were captured at a magnification of ×40 with a bar length of 50μm. All values are presented as mean ± SE (n = 6). Statistical analysis was performed by unpaired t test, **p < 0.01, ***p < 0.001, #p < 0.05, ^##^p < 0.005, ^###^ p < 0.001 **(B**-(3)). **(C)** Expression of cytokines and chemokines in peritoneal disseminated tumors was assessed by real-time RT-PCR in mice inoculated with Panc02 cells overexpressing ZG16 or lacking programmed cell death ligand 1 (PD-L1) gene, as well as control Panc02- inoculated mice. The data presented are mean ± SE (n = 6). Statistical analysis was performed using one –way ANOVA. Statistical significance is denoted as *** p < 0.001, **** p <0.0001. **(D)** Cytokine expression in the ascites of PD-L1 knockout, ZG16 overexpression, and control animals was assessed. The data presented are mean ± SE (n = 6). Statistical analysis was performed using one –way ANOVA. Statistical significance is denoted as, ** p < 0.01, *** p < 0.001, **** p <0.0001.

To verify the efficacy of PD-L1 knockout and ZG16 expression *in vivo*, mice were injected with PD-L1 knockout, ZG16 overexpression and control Panc02 cells to establish an intraperitoneal metastasis model. Mice were sacrificed 14 days post the initial injection, and the tumors was harvested and analyzed. Tumor weight and ascites volume exhibited significant reductions in the PD-L1-KO and ZG16-inoculated groups compared to the control Panc02-inoculated group (**Figure 6A**-(1-3)). These findings suggest that genetic disruption of tumor-derived PD-L1 through gene knock-down and ZG16 overexpression may effectively impede intraperitoneal tumor progression.

### 3.7 Interference with PD-L1 and increased ZG16 expression affect the infiltration of tumor lymphocytes *in vivo*

To elucidate the influence of PD-L1 disruption and ZG16 overexpression on inhibiting PDAC progression, we conducted immunohistochemical analysis and profiled tumor-infiltrating lymphocytes. The quantities of CD4+ T cells, CD8+ T cells, and CD49+ NK cells within the tumors were significantly elevated in both the PD-L1-KO and ZG16 groups compared to their respective control groups (**Figure 6A**-(2)). Moreover, a significant decrease in the number of PD-L1+, CTLA4+, and PD-1+ cells within the tumor microenvironment was observed in both the PD-L1-KO group and the ZG16 group when compared to the control group (**Figure 6A**-(2)).

### 3.8 Identification of macrophage subpopulations in *in vivo* tumor tissues using double-color immunofluorescence analysis

In the Panc02 control group, a majority of intratumor F480+ macrophages were CD206-positive M2 macrophages (**Figure 6B**-(1, 3)). Conversely, in the shPD-L1 (PD-L1-KO) and ZG16 high expression groups, most intratumor F480+ macrophages exhibited CD11c positivity indicative of M1 polarization (**Figure 6B**-(1,3)). Furthermore, F480+ macrophages in the PD-L1-KO and ZG16-overexpressed Panc02 vaccination groups expressed CXCL9 and CXCl10 chemokines responsible for recruiting CD8+ T cells (**Figure 6B**-(2,3)). These findings suggest that PD-L1 disruption on tumor cells and ZG16 overexpression induce recruitment of anti-tumor immune cells. Additionally, they promote an increase in M1 macrophage population rather than M2 phenotype while upregulating production of CXCL9 and CXCl10 by these macrophages, thereby facilitating infiltration of CD8+ killer T cells into the tumor microenvironment. Such alterations in immune cell profile may contribute to suppression of PDAC progression.

### 3.9 The influence of PD-L1 disruption and ZG16 overexpression on the levels of cytokines and chemokines in mouse ascites and tumors

The results demonstrated significantly higher mRNA expression of IFN-γ, TNF-α, IL-2, IL-12a, CXCL9, and CXCL10 in the tumor tissue of the PD-L1-KO/ZG16 overexpression group compared to the control group (**Figure 6C**). Conversely, expression levels of IL-10, VEGF, CXCL1, and CXCL2 were significantly lower than those in the control group (**Figure 6C**). Additionally, both IL-10 and VEGF levels in ascites from the PD-L1 deficient/ZG16 overexpression group were markedly reduced compared to the control group (**Figure 6D**). Consistent with these findings in tumor tissue samples, elevated levels of IFN-γ and TNF-α were also observed in ascites from this experimental group when compared to controls (**Figure 6D**). These results suggest that disrupting PD-L1 expression in PDAC cells while up-regulating ZG16 can enhance immune activation by modulating cytokines/chemokines involved while simultaneously down-regulating immunosuppressive cytokines as well as tumor angiogenic factors.

## 4 Discussion

We found that the loss of PD-L1 in Panc-1 PDAC cells inhibited cell growth, proliferation, and migration, and also led to the polarization of TAMs from M2 to M1. Targeting mouse PD-L1 using mouse LV-shPD-L1 *in vivo* also causes macrophages to polarize from M2 to M1.

PD-L1 was highly expressed in the cytoplasm and nucleus of Panc-1 cells. The proliferation, growth, invasion and migration of Panc-1 cells were inhibited by down-regulating the expression of PD-L1 in the cytoplasm and nucleus of Panc-1 cells. Consistent with the results of other studies, PD-L1 is important for tumor growth and progression (15).

After PD-L1 knockdown in Panc-1 cells, the polarized TAMs changed from M2 to M1. The inhibitory effect of PD-L1 may be a direct therapeutic strategy against PDAC. In an *in vitro* PDAC tumor setting, Cas9 + hdr treated Panc-1 cells resulted in decreased M2 TAM polarization, increased M1 TAM polarization, PD-L1 depletion inhibited M2 IL-4 secretion, and upregulated M1 TNF-αsecretion. Our results suggest that the loss of PD-L1 in PDAC cells promotes immune activation in the tumor microenvironment. It was further found that PD-L1 knockout could polarize mouse macrophages. Cas9 dual sgrna + HDR system inhibited the expression of PD-L1 protein on the cell surface and intracellular.

We have also demonstrated that the overexpression of ZG16 in the human PDAC cell line Panc-1 can achieve a tumor immunomodulatory effect comparable to that of PD-L1 knockout. Specifically, ZG16 overexpression promotes the polarization of TAMs from M2 to M1 type, inhibits the proliferation, growth, invasion, and migration of Panc-1 cells, enhances immune activation within the tumor microenvironment *in vivo*, and also mitigates immune resistance in mouse PDAC.

In the tumor microenvironment, various immunosuppressive mechanisms may be activated, thereby hindering effective anti-tumor immunity (20). Immune checkpoint inhibitors target immune checkpoints such as PD-1/PD-L1 and CTLA-4, and promote survival through cytotoxic T cell activity. It has been demonstrated that this is an important pathway associated with various malignant tumors, including pancreatic ductal adenocarcinoma (PDAC). Various types of mutations, signaling pathways, immune system-based therapeutic approaches, and potential therapeutic targets for pancreatic ductal adenocarcinoma (21).

For instance, point mutations in K-Ras serve as an archetypal genetic variation that is prevalent in patients with pancreatic ductal adenocarcinoma (PDAC) (22). Originating from early pancreatic diseases such as low-grade pancreatic intraepithelial neoplasms (PanINs), it is a slowly progressing, invasive condition. A large number of downstream effectors are implicated in K - Ras signaling, such as the classic Raf/MAPK/extracellular signal - regulated kinase (Erk), PI3Ks/(PDK - 1)/Akt, RalGEF and phospholipase Cϵ, etc. (23). Just as the KEGG signaling pathways we enriched in this study, such as the Wnt/β - catenin signaling pathway, the mitogen - activated protein kinase (MAPK) signaling pathway, etc.

For another example, the expression of constitutively active oncogenic class 1A PI3K (PI3CA H1047R) in Ptf1a-positive cells can induce acinar-to-ductal metaplasia. Key signal transducers of the precancerous PanIN signaling pathway are inactivated in more than half of pancreatic cancer patients. Regarding the signaling of growth factor receptors, several mitogenic growth factors and their ligands are overexpressed in pancreatic cancer, such as: EGF and EGFR (the receptor of EGF), multiple EGFR-binding ligands; IGF and its receptor (IGFR); platelet-derived growth factor; and vascular endothelial growth factor (24). As well as the KEGG signaling pathways enriched in our study: epidermal growth factor receptor tyrosine kinase inhibitor resistance, ErbB signaling pathway, etc.

In addition, EMT plays a crucial role in the rapid progression of pancreatic cancer tumors. During EMT progression, epithelial cells lose markers such as E-cadherin, occludin, claudin, and plakoglobin 1 (epithelial markers), and acquire N-cadherin, vimentin and fibronectin (mesenchymal markers) (25). This is consistent with the KEGG signaling pathways we have enriched in this study: the chemokine signaling pathway; and the differentially expressed gene VIM (Vimentin). At the same time, VIM is also a common gene in PD-L1 knockout and ZG16 overexpression.

Clinical trials have shown that targeted molecules such as CTLA-4 and PD-1 in cancer patients can restore the anti-tumor immune response. The immune-suppressive activity of PD-L1 is tightly regulated by ubiquitination and N-glycosylation. Glycogen synthase kinase 3b (GSK3b) interacts with PD-L1 and induces ubiquitin-dependent proteasomal degradation of PD-L1 through the E3 ubiquitin ligase b-TrCP. Glycosylation antagonizes GSK3b binding. Epidermal growth factor (EGF) stabilizes PD-L1 through GSK3b inactivation in basal-like breast cancer. Meanwhile, the epidermal growth factor (EGF) stabilizes PD-L1 through GSK3b inactivation in basal-like breast cancer (26). As we have enriched in this study, KEGG signaling pathways such as: glycosaminoglycan biosynthesis-chondroitin sulfate/dermatan sulfate; glycosphingolipid biosynthesis-globo series and isoglobo series; glycosylphosphatidylinositol (GPI) anchor biosynthesis; mannose-type O-linked glycosylation; N-linked glycosylation; proteoglycans in cancer.

TME (tumor microenvironment) is an obstacle to immunotherapy strategies. Tumor-infiltrating lymphocytes (TILs) (27), a genetically diverse population of immune cells associated with TME, including CD8+ T lymphocytes and CD4+ T1 lymphocytes, have been linked with favorable outcomes. In contrast, CD4+ T2 lymphocytes have a detrimental impact on patient survival. Immune cells and inflammatory cells, as well as polarized macrophages, are fundamental components of the pancreatic cancer TME and contribute to chemoresistance. These cells play a role in fibrosis and neovascularization (25). As also enriched in this study, KEGG pathways associated with immune regulation such as: Amyotrophic Lateral Sclerosis, Autoimmune Thyroid Disease, and B Cell Receptor Signaling Pathway, as well as TAM-related pathways. Significantly different genes found in sequencing analysis, including cancer driver genes such as RET/DDIT3/ITGB8/COL6A3/cxcl2, oncogenes such as fos, WNT family genes, immune dysregulation oncogenes such as SYK, innate immune response genes TLR3, and pan-cancer new target genes such as NRG1 gene fusion, IL2RB joint immunodeficiency-related genes, and NTRK1 tumor driver genes, etc. Among them, NEURL1, DDIT3,VIM,TEX19,PRSS22,CNTNAP2 are the common differentially expressed genes caused by PD-L1 knockout and ZG16 overexpression.

The FUS-DDIT3 fusion gene is recognized as the driver gene of myxoid liposarcoma (MLS). The evidence supporting its contribution to MLS includes the following: Firstly, FUS serves as a downstream target of ATM, a key regulator of DNA repair, and plays an active role in DNA damage repair processes. Secondly, DDIT3 has been shown to inhibit adipocyte differentiation by binding to members of the CEBP-β protein family, which are principal regulators of adipogenesis (28). Thirdly, mesenchymal stem cells expressing FUS-DDIT3 rely on YAP1 (29). It’s truly remarkable to note that the biological behaviors of the genes associated with DDIT3 discovered in this study exhibited opposite patterns in two distinct cell types (PD-L1 knockout and ZG16 overexpression). It’s essential to conduct further research to determine if there exists a specific intrinsic genetic driving mechanism that gives rise to this biological expression and the potential biological consequences that might result from it.

In solid tumors, PDAC is one of the most immunologically resistant tumor types, an immunologically ‘cold tumor’, a key feature of which is an extreme lack of CD8+ T cells, leading to immune surveillance evasion and a low response rate to immune checkpoint antibodies (30). Although single-agent immunomodulatory drugs have had limited clinical efficacy in PDAC (31), exploring multimodal therapies that target mechanisms of resistance to immunotherapy may have significant implications for the treatment of PDAC. Therefore, developing new immunotherapeutic regimens to activate the PDAC immune pathway, transforming it from an immunologically tolerant to an immunologically activated state, may serve as a new therapeutic strategy for PDAC.

The author’s prior research has demonstrated that ZG16 can directly interact with glycosylated PD-L1 via its lectin structure, thereby enhancing local tumor immunity (18, 32). Overexpression of ZG16 in colon cancer cells significantly influences the expression of both stimulatory and inhibitory checkpoint molecules, including CD40, PD1, and CTLA4 (32). Furthermore, experimental results have confirmed that the sustained *in situ* activation induced by ZG16 system injection on CD40, along with the role of ZG16 in conjunction with dendritic cells (DCs) to enhance their function, may offer a novel research avenue for immunotherapy targeting pancreatic ductal adenocarcinoma (PDAC). However, the specific mechanism of action remains to be further elucidated (17).

CD40 agonists can enhance the expression of costimulatory and antigen presentation molecules on macrophages, promote the secretion of pro-inflammatory mediators, and enhance T-cell-dependent antitumor effects (19). It is known that TLS (Toll-like receptor signal) and CD40 can be activated by IFN-γ (33, 34), which is also the driving force of M1 polarization. In addition, PD-L1 induces an immunosuppressive phenotype and suppresses antigen presentation ability by reducing the expression of costimulatory molecules in macrophages. PD-L1 blockade increases the generation of costimulatory molecules (CD86 and MHC-II) and pro-inflammatory cytokines (tumor necrosis factor A [TNFα] and IL12), including the phenotype and expression characteristics of M1 macrophages (35). Therefore, we hypothesize that ZG16 may further modulate macrophage polarization by influencing the expression of both stimulatory and inhibitory checkpoint molecules, including CD40, PD1, and CTLA4. Additional data are required to substantiate this hypothesis.

Furthermore, both PD-L1 knockout and ZG16 overexpression in cancer cells can target PD-L1 and CTLA4 within the tumor immune microenvironment. This finding offers new insights and methodologies for Panc-1 treatment and holds promise for significant breakthroughs in clinical applications.

## 5 Conclusions

The knockout of PD-L1 or intervention with ZG16 can effectively inhibit the proliferation and invasion of PDAC cells, as well as influence the polarization of tumor-associated macrophages. This discovery offers a novel approach for the treatment of PDAC.

## Data Availability

The data presented in the study have been deposited in the SRA repository, accession number: PRJNA1216073 (https://www.ncbi.nlm.nih.gov/sra/PRJNA1216073).

## Supplementary material

Supplementary Figure 1 Flowchart of the Overall Experimental Approach and Methodology
