# Supplementary figures and images for: PD-L1 knockout or ZG16 overexpression inhibits PDAC progression and modulates TAM polarization
Source: Front Immunol. 2025 Jan 31;16:1510179. doi: 10.3389/fimmu.2025.1510179 (PMC11826313; doi:10.3389/fimmu.2025.1510179)

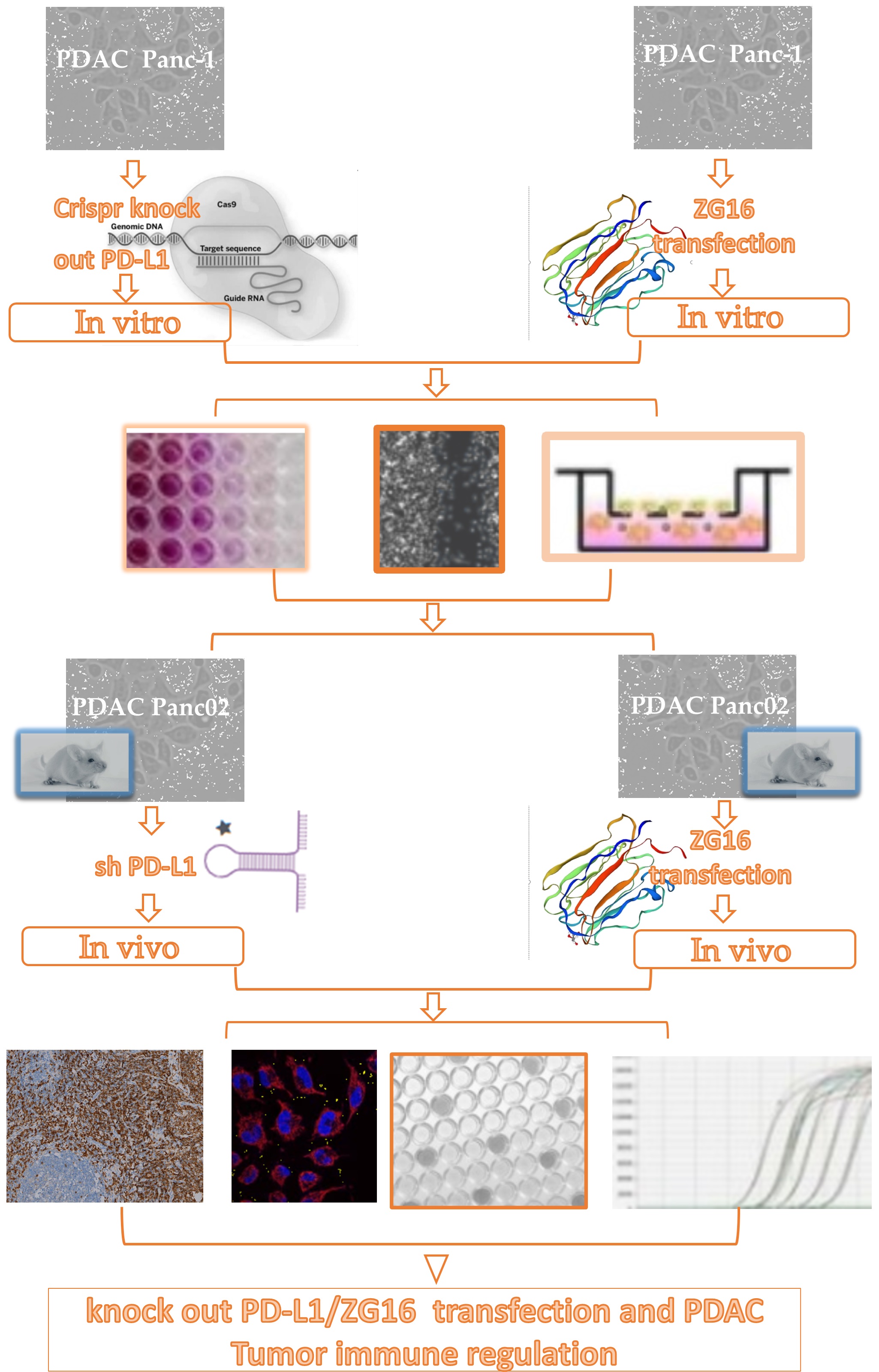

Supplement: Supplementary Figure 1 — Flowchart of the Overall Experimental Approach and Methodology [file Image1.jpeg]
